# Cone beam CT-based adaptive intensity modulated proton therapy assessment using automated planning for head-and-neck cancer

**DOI:** 10.1186/s13014-024-02406-9

**Published:** 2024-01-23

**Authors:** Yihang Xu, William Jin, Michael Butkus, Mariluz De Ornelas, Jonathan Cyriac, Matthew T. Studenski, Kyle Padgett, Garrett Simpson, Stuart Samuels, Michael Samuels, Nesrin Dogan

**Affiliations:** 1 Department of Radiation Oncology, University of Miami Miller School of Medicine, Miami, FL USA; 2 Department of Biomedical Engineering, College of Engineering, University of Miami, Coral Gables, FL USA; 3 Department of Radiation Oncology, Banner MD Anderson Cancer Center, Gilbert, AZ USA

**Keywords:** Adaptive, IMPT, Head and neck cancer, CBCT

## Abstract

**Background:**

To assess the feasibility of CBCT-based adaptive intensity modulated proton therapy (IMPT) using automated planning for treatment of head and neck (HN) cancers.

**Methods:**

Twenty HN cancer patients who received radiotherapy and had pretreatment CBCTs were included in this study. Initial IMPT plans were created using automated planning software for all patients. Synthetic CTs (sCT) were then created by deforming the planning CT (pCT) to the pretreatment CBCTs. To assess dose calculation accuracy on sCTs, repeat CTs (rCTs) were deformed to the pretreatment CBCT obtained on the same day to create deformed rCT (rCT_def_), serving as gold standard. The dose recalculated on sCT and on rCT_def_ were compared by using Gamma analysis. The accuracy of DIR generated contours was also assessed. To explore the potential benefits of adaptive IMPT, two sets of plans were created for each patient, a non-adapted IMPT plan and an adapted IMPT plan calculated on weekly sCT images. The weekly doses for non-adaptive and adaptive IMPT plans were accumulated on the pCT, and the accumulated dosimetric parameters of two sets were compared.

**Results:**

Gamma analysis of the dose recalculated on sCT and rCT_def_ resulted in a passing rate of 97.9% ± 1.7% using 3 mm/3% criteria. With the physician-corrected contours on the sCT, the dose deviation range of using sCT to estimate mean dose for the most organ at risk (OARs) can be reduced to (− 2.37%, 2.19%) as compared to rCT_def_, while for V95 of primary or secondary CTVs, the deviation can be controlled within (− 1.09%, 0.29%). Comparison of the accumulated doses from the adaptive planning against the non-adaptive plans reduced mean dose to constrictors (− 1.42 Gy ± 2.79 Gy) and larynx (− 2.58 Gy ± 3.09 Gy). The reductions result in statistically significant reductions in the normal tissue complication probability (NTCP) of larynx edema by 7.52% ± 13.59%. 4.5% of primary CTVs, 4.1% of secondary CTVs, and 26.8% tertiary CTVs didn’t meet the V_95_ > 95% constraint on non-adapted IMPT plans. All adaptive plans were able to meet the coverage constraint.

**Conclusion:**

sCTs can be a useful tool for accurate proton dose calculation. Adaptive IMPT resulted in better CTV coverage, OAR sparing and lower NTCP for some OARs as compared with non-adaptive IMPT.

## Background

Radiation therapy can serve as a primary or adjuvant treatment to surgery for head-and-neck (HN) cancer patients. Both intensity-modulated radiation therapy (IMRT) and intensity-modulated proton therapy (IMPT) are well suited for the complex anatomy in HN cancer treatments as they deliver highly conformal dose to tumor volumes while sparing organs at risk (OAR). IMRT is a common treatment technique, but studies have demonstrated that IMPT can deliver a superior dose distribution compared to IMRT due to the physical property of proton dose deposition known as the “Bragg peak” [1, 2]. However, IMPT dose delivery is very sensitive to anatomical variations, setup deviations, and range uncertainties due to Hounsfield unit (HU) to stopping power (SP) conversion. These geometric and positional changes can result in OAR toxicity and under dosage to the clinical target volumes (CTV). Additionally, significant inter-fractional anatomic changes due to weight loss commonly occur during HN treatment [3–5]. One technique for managing these changes is to adapt the treatment plan to the new anatomy so an optimal dose distribution can be maintained throughout the treatment course.

Traditional offline adaptive radiation therapy (ART) typically requires an acquisition of repeat CT (rCT) scans during the treatment course. Acquisition of rCTs contributes extra imaging dose to the patient, requires additional departmental resources, and might provide a false indication for adaptation if the patient setup is not done carefully [6]. In image-guided radiation therapy (IGRT), pretreatment cone-beam computed tomography (CBCT) images are becoming a standard of care for patient setup. CBCT images acquired during the standard care path require fewer resources and reduce the overall imaging dose burden to the patient compared to acquiring additional rCTs. However, it is not recommended to use CBCTs directly for dose calculations due to poor image quality caused by increased scatter, motion, beam hardening, and other imaging artifacts, especially for proton therapy [7–10]. Despite these limitations, early studies exploring the use of CBCTs for accurate proton dose calculations show promise [7–19].

Attempts have been made to reduce the uncertainties in using CBCTs for ART. Scatter correction on CBCT has been studied by several groups as a reliable method for accurate proton dose calculation [7, 9, 10]. Another approach is using deformable image registration (DIR) to transfer the HU information from the planning CT (pCT) to the CBCT to create a synthetic CT (sCT) [10–15]. Work by Kurz et al. compared proton doses recalculated on sCT and scatter corrected CBCT and found high dosimetric agreement for both head and neck and prostate treatments [10].

The potential benefits of adaptive proton therapy (APT) have been reported by several groups. Simone et al. compared the APT and non-APT using rCT for HN patients and indicated that the APT improves OAR sparing [1]. Another study by Gora et al. compared dosimetric benefits between photon ART and APT for six HN patients and found that APT plans improved target coverage and OAR sparing for brainstem and spinal cord [25]. Lalonde et al. compared robustly optimized IMPT plans to daily adaptive IMPT without robustness constraints for ten HN patients and concluded that daily adaptation resulted in better target coverage and OAR sparing [22]. Botas et al. investigated the feasibility of a fast weight-tune online adaption approach based on Monte Carlo methods with CBCT and indicated significant improvements in plan quality [26]. Nenoff et al. studied the benefits of APT by simulating different nasal cavity filling and setup scenarios for five paranasal patients, concluding that APT improves plan robustness [21]. More recently, an investigation by Borderías-Villarroel et al. explored the dose difference between non-APT and APT based on different strategies (manually full re-optimization or automatic isodose volume dose restoration) for ten HN patients. Results demonstrated that the dose restoration method could achieve CTV coverage for half of the patients, but manual full re-optimization was still required for the other patients [23]. Bobic et al. compared the daily adaptation and weekly adaptation using another dose restoration method and recommended weekly adaptation achieved satisfactory CTV coverage for most patients [27]. While these studies have demonstrated the potential dosimetric benefits of APT with small sample sizes, the potential clinical relevance of these dosimetric benefits has not been explored.

Daily online dose evaluation and plan adaptation requires a fast turnaround to be clinically feasible. In an ART workflow, the contouring and re-optimization steps present a challenge as they are resource intensive, but DIR presents an option to quickly transform contours from the pCT to the daily image. Previous studies have demonstrated the feasibility of using DIR-based sCT for proton dose calculation in HN region via stopping power (SP) comparison, water equivalent thickness (WET) comparison, or gamma analysis between sCT and reference CT [10, 12–15], but only a few studies investigated the impact of uncertainty of DIR-propagated contours on the dose evaluation or plan adaptation in HN region [15, 20].

In terms of the proton plan adaptation, previous studies investigated re-optimization with the same objective list [21, 22], manual full re-optimization [1, 23], or the fast dose restoration method [23, 24] to generate a new plan on the daily image. However, manual full re-optimization is time consuming and dose restoration or re-optimization with initial objective list might not result in the optimal dose distribution when large anatomical changes occur [23]. Our previous study investigating an automated planning software model for efficient HN IMPT plan generation demonstrated the model-generated plans have a quality that is, at minimum, comparable to the manual plans produced by dosimetrists [28].

The primary goals of this study are to quantify the accuracy of proton dose calculation using DIR-based sCT and the impact of DIR propagated contour inaccuracy on dosimetric evaluation. We also aim to quantify the dosimetric benefits of an efficient offline adaptive IMPT workflow using weekly CBCT and a pre-validated automated planning software [28]. This study utilizes the automated planning system for plan adaptation to ensure the optimal dose distribution and reduce the inter-operator variation during re-optimization. The clinical significance of the dosimetric benefits will be estimated by employing radiobiological modeling.

## Methods

### Patient cohort and IMPT planning

Twenty patients with advanced HN cancer previously treated with IMPT or VMAT were included in this study. All patients were enrolled in a retrospective institutional review board (IRB) approved protocol. For each patient, contrast and non-contrast pCTs were acquired on the same day in a supine position with 2-mm slice thickness using the Siemens Somatom CT 16 slice or 64 slice simulators (Siemens Healthineers AG, Germany). All gross tumor volumes (GTVs), clinical target volumes (CTVs), and OARs (spinal cord, brainstem, parotids, constrictors, mandible, cochlea, larynx, carotids, oral cavity, submandibular, eyes, and optic nerves) were delineated on the contrast CT. The volumes were then rigidly transferred to the non-contrast CT. Patients were prescribed one to three dose levels ranging from 56 to 70 Gy delivered in 30–35 fractions. Table 1 shows the characteristics of the patients included in this study. The CTVs were located in the mid/lower neck area for a vast majority of patients in this study. A daily pretreatment CBCT scan for setup was obtained with a ProBeam compact or TrueBeam on-board CBCT imager (Varian Medical System, Inc, Palo Alto, California). Ten patients had at least one rCT scan using the same protocol as their pCT during their treatment course.

**Table 1:** Patient characteristics

Subsite	T stage	Treatment modality	Prescribed dose (Gy)	Number of fractions	Treatment intent	Fraction number when rCT is acquired
Nasopharynx	T1N1	IMPT	70, 59.5, 56	35	Definitive chemoradiation	17, 27
Parotid	T1N0	IMPT	60	30	Adjuvant radiation	10
Oral cavity	TxN3	VMAT	70, 63, 56	35	Re-irradiation	13
Oropharynx	T2N2	VMAT	70, 59.5, 56	35	Definitive chemoradiation	18
Oropharynx	T2N3	VMAT	70, 60, 56	35	Definitive chemoradiation	NA
Oropharynx	T1N2	VMAT	70, 59.5, 56	35	Definitive chemoradiation	NA
Oropharynx	T2N1	VMAT	70, 60, 56	35	Definitive chemoradiation	NA
Oropharynx	T2N3	VMAT	70, 60, 56	35	Definitive chemoradiation	NA
Oropharynx	T2N1	VMAT	70, 60, 56	35	Definitive chemoradiation	NA
Larynx	T4aN2c	VMAT	70, 63, 56	35	Definitive chemoradiation	NA
Hypopharynx	T1N3	IMPT	70, 63, 56	35	Definitive chemoradiation	2
Oropharynx	T3N1	VMAT	70, 59.5, 56	35	Definitive chemoradiation	NA
Larynx	T4aN2c	VMAT	70, 63, 56	35	Definitive chemoradiation	NA
Peripheral nerve	T1N0	IMPT	60	30	Adjuvant radiation	NA
Nasopharynx	T2N2	IMPT	70, 59.5, 56	35	Definitive chemoradiation	23
Parotid	T2N0	IMPT	60	30	Adjuvant radiation	22
Parotid	T2N0	IMPT	66	33	Adjuvant radiation	7, 12, 20
Oropharynx	TxN3	IMPT	66, 59.4, 56.1	33	Salvage chemoradiation	4
Nasopharynx	T4aN0	IMPT	66	33	Adjuvant chemoradiation	16
Oral cavity	T4aN0	IMPT	60	30	Salvage radiation	NA

### Initial IMPT plan generation

IMPT plans were generated for each patient using a pre-validated automatic planning software (RapidPlanPT, version 16.2, Varian Medical Systems, Palo Alto, CA) model with multifield optimization (MFO) to reduce the inter-operator variability [28]. RapidPlanPT (RPP) employs a dose-volume histogram (DVH) estimation model trained from a library of high-quality treatment plans. The dose-volume objectives were automatically placed near the lower boundary of each OAR DVH prediction range to guide the optimization process [28]. The beam number and arrangements were selected based on tumor anatomy and location and field-specific targets were created for each field. Proton spots could only be placed in the field-specific targets that were created to encompass all CTVs including 3 mm positional setup uncertainty and 3% range uncertainty for each field [29]. The field-specific targets were modified to prevent beams entering through the chin and teeth area to reduce proton range uncertainty from the movement of the chin or tongue. Streaking artifacts caused by dental implants were delineated and overridden to an HU value approximate to the surrounding soft tissue.

The non-linear universal proton optimizer (NUPO 16.02, Eclipse, Varian Medical Systems) was utilized for optimization along with the proton convolution superposition algorithm for dose calculation (PCS 16.02, Eclipse, Varian Medical Systems). A 2 mm × 2 mm × 2 mm dose grid was used along with a relative biological effectiveness (RBE) of 1.1 to weight the dose. All IMPT plans were robustly optimized using ± 3 mm setup uncertainty (in cardinal directions) along with ± 3% proton range uncertainty, resulting in 12 uncertainty scenarios. The targets were the only structures selected to be robustly optimized. Plans were optimized using an autogenerated objective list by the RPP model. If needed, one to two additional optimization iterations were performed to improve the CTV coverage and OAR sparing using a fine-tune of the objective list. These additional optimizations for some patients included satisfying the CTV coverage, reducing target maximum dose, or slightly improving OAR sparing if the clinical constraint was not met. All IMPT plans were normalized such that 95% of the primary CTV volume was covered by 100% of the prescription dose (V100 = 95%). Robust evaluation was performed by introducing the same uncertainty as in the robust optimization. The worst-case scenario in the robust evaluation required all CTVs to achieve at least 95% of each volume receiving 95% of the prescribed dose (V95 > 95%). The dose-volume constraints for OARs are given in Table 2.

**Table 2:** Dose constraints for the OARs for IMPT planning

Structures	Dose-volume constraints
Body	Dmax < 115%
Brainstem	Dmax < 54 Gy
Cochlea	Dmean < 40 Gy
Pharynx constrictor	Dmean < 50 Gy
Larynx	Dmean < 50 Gy
Mandible	Dmax < 75 Gy
Oral cavity	Dmean < 50 Gy
Spinal cord	Dmax < 48 Gy
Parotid	Dmean < 26 Gy
Parotid	V20Gy < 50%
Esophagus	Dmean < 40 Gy
Eye	Dmax < 45 Gy
Optic nerve	Dmax < 50 Gy
Optic chiasm	Dmax < 50 Gy
Submandibular	Dmean < 39 Gy

### Validation of proton dose calculation based on sCT

**Fig. 1:**
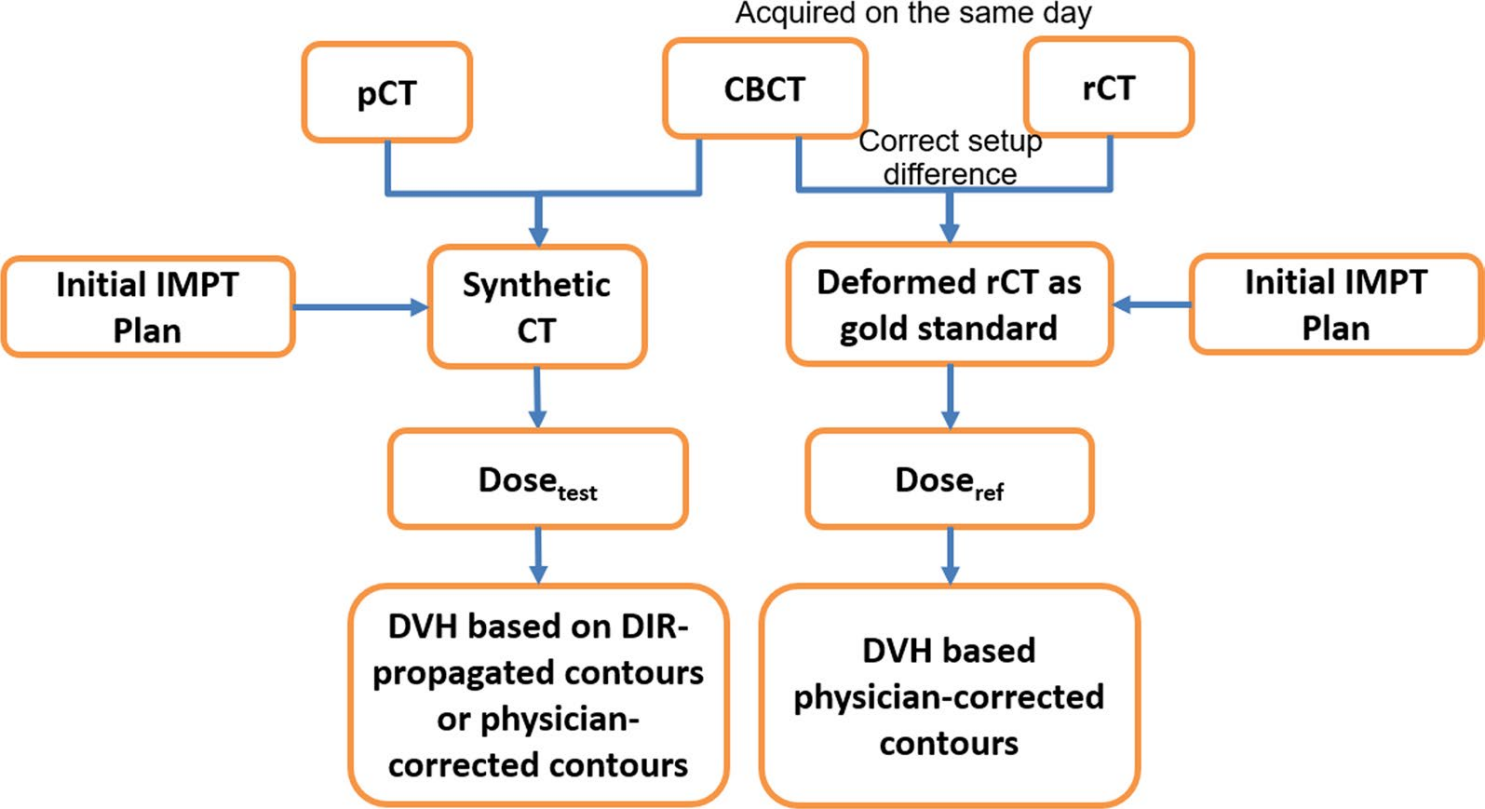
Workflow for the validation of CBCT-based proton dose calculation on the sCT

Thirteen rCTs from ten patients were used to validate the accuracy of proton dose calculation on the sCT. The workflow of the validation is shown in Fig. 1. All the data were imported into an imaging software package (Velocity, version 4.1, Varian Medical Systems, Palo Alto, CA) that contains a B-spline based DIR with a built-in CBCT correction algorithm. For sCT generation, the daily CBCT acquired on the same day as the rCT was rigidly registered to pCT by applying the shifts from the patient setup. A displacement vector field (DVF) was then calculated through DIR between the pCT and daily CBCT. The DVF was used to create a sCT by transferring the HU and overridden HU values (e.g., dental artifact) from the pCT to the CBCT frame of reference. Areas outside the CBCT field-of-view (FOV) were filled with the corresponding image data from the pCT source image.

Visual assessment of the DVF was performed to ensure that the DIR transformations were physically and anatomically reasonable. Anatomical landmarks including bony anatomy and the body contour were confirmed to match in the images. Additional refinement was rarely needed to improve DIR accuracy, and this was accomplished by adjusting the region of interest to focus on a smaller region around the CTVs. Contours from the pCT were transformed to the sCT using the same DVF, as well. Although the DVFs appeared visually accurate, additional corrections to the DIR-propagated contours might be required by a physician based on their review. In these cases, two contour sets were created on the sCT—purely DIR propagated contours and physician-corrected contours. The physician-corrected contours served as the gold standard.

Finally, the rCT was deformably registered to the daily pretreatment CBCT. The resulting deformed rCT (rCT_def_) served as the gold standard for proton dose calculation as this image provided true HU values in the same frame of reference as the sCT. The gold standard physician-corrected contours from the sCT were rigidly transferred to the rCT_def_.

The initial IMPT plan was recalculated on both the sCT and rCT_def_. To validate the accuracy of proton dose calculation on the sCT, 3D global gamma analysis [30] with 3 mm/3% and 2 mm/2% was performed within the region receiving > 10% of the prescribed dose. The gamma analysis was performed in MATLAB R2022b (MathWorks Inc. Natick, MA).

### Validation of DIR contour propagation

To investigate the impact of DIR propagated contour inaccuracy (including SP transfer uncertainty) on dosimetric evaluation, the dose from the initial IMPT plan was also calculated on the sCT with uncorrected DIR propagated contours (sCT_uncorr).The dose volume indices from the sCT_uncorr were then compared to the indices from the gold standard rCT_def_ with physician-corrected contours. Additionally, the dice similarity coefficient (DSC) was calculated for the CTVs and OARs to quantify the agreement between the DIR propagated (uncorrected) and physician-corrected volumes.

### Benefit of adaptation versus non-adaptation

Twenty patients with daily CBCTs were included in the assessment of offline APT versus non-APT. Previously published work demonstrated that weekly CBCTs accurately estimate and represent the overall delivered dose to HN cancer patients [3]. Therefore, daily pretreatment CBCTs were selected every five fractions (e.g., fraction 1, 6, 11, 16, etc.) to serve as weekly images and were used to create weekly sCTs.

For the non-adaptation (non-adapt) group, the proton doses were recalculated on the weekly sCTs using the initial IMPT plan. The dose accumulation was performed by warping the weekly recalculated dose to the pCT through the inverse DVF that was used to create the sCTs. For the adapted (Adapt) group, the IMPT plans were reoptimized on each weekly sCT using the automated planning software RPP [28]. Plans were optimized with the same robustness parameters used in the initial plan and dose accumulation was performed using the same method as for the non-adapt group. The weekly dosimetric parameters for CTV and OARs were compared between the non-adapt and adapt groups. The percentage of fractions that met the dose constraints from Table 2 were calculated for each group. Next, the accumulated dose-volume indices from non-Adapt and Adapt patients were evaluated. Finally, normal tissue complication probability (NTCP) models were employed to estimate the probability of > grade 2 larynx edema, > grade 2 dysphagia, > grade 4 xerostomia, and > grade 2 acute esophagitis following the end of the treatment course [31–34]. All statistical analysis was performed using a two-sided paired t-test in JMP Pro (SAS Institute Inc.). A *P* value < 0.05 indicated a significant difference.

## Results

### Validation of proton dose evaluation based on sCT

**Fig. 2:**
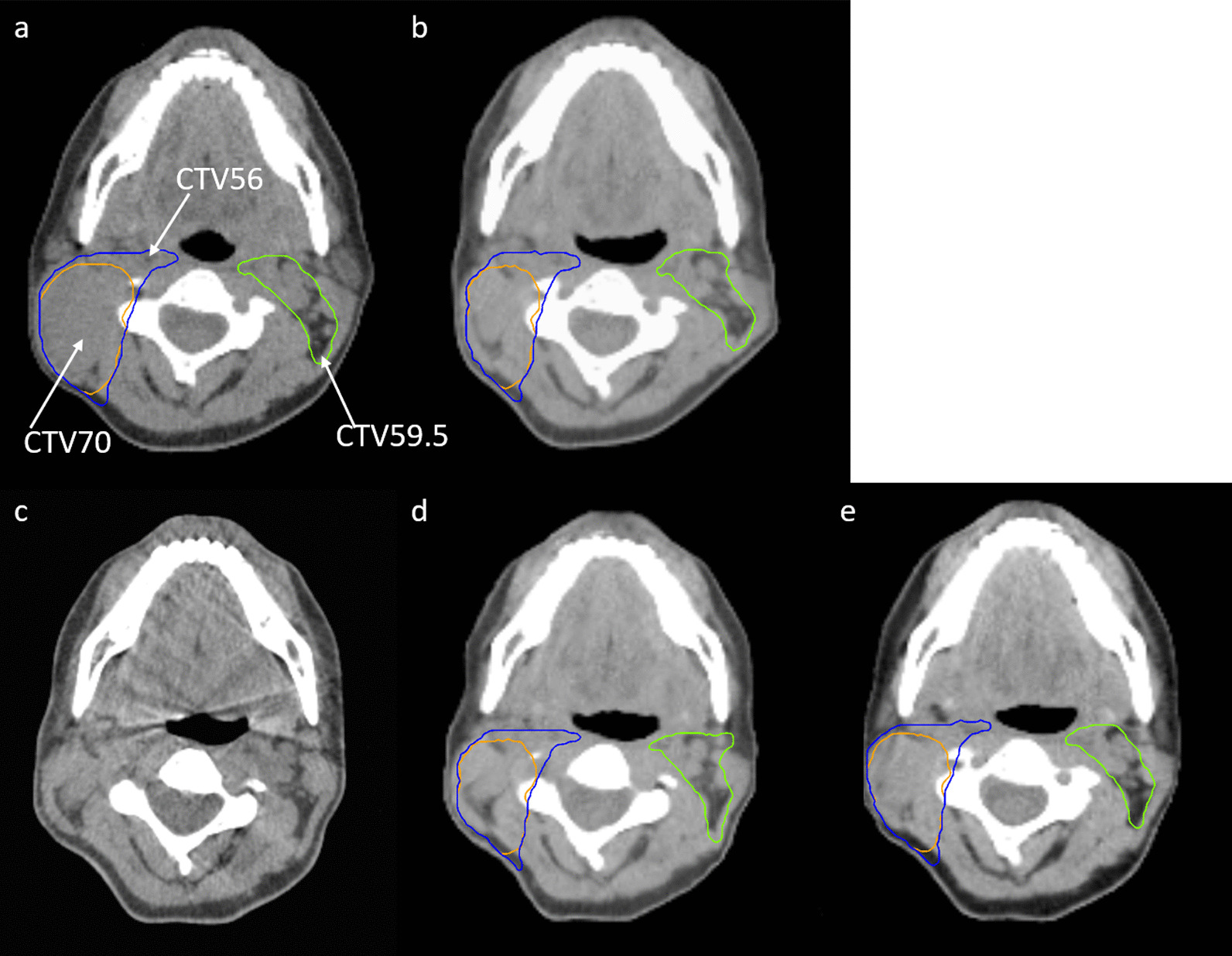
An example case with different CT images. **a** pCT; **b** rCT; **c** CBCT; **d** rCT_def_ with gold standard contours; **e** sCT with DIR-propagated contours

An example case with different pCT, rCT, CBCT, rCT_def_ and sCT is provided in Fig. 2. High dosimetric agreement was observed between the dose calculated on the rCT_def_ (gold standard) and sCT with a mean gamma pass rate of 97.9% ± 1.7% for 3 mm/3% criteria and 93.7% ± 4.2% for 2 mm/2% criteria throughout the whole body.

### Validation of DIR contour propagation

**Fig. 3:**
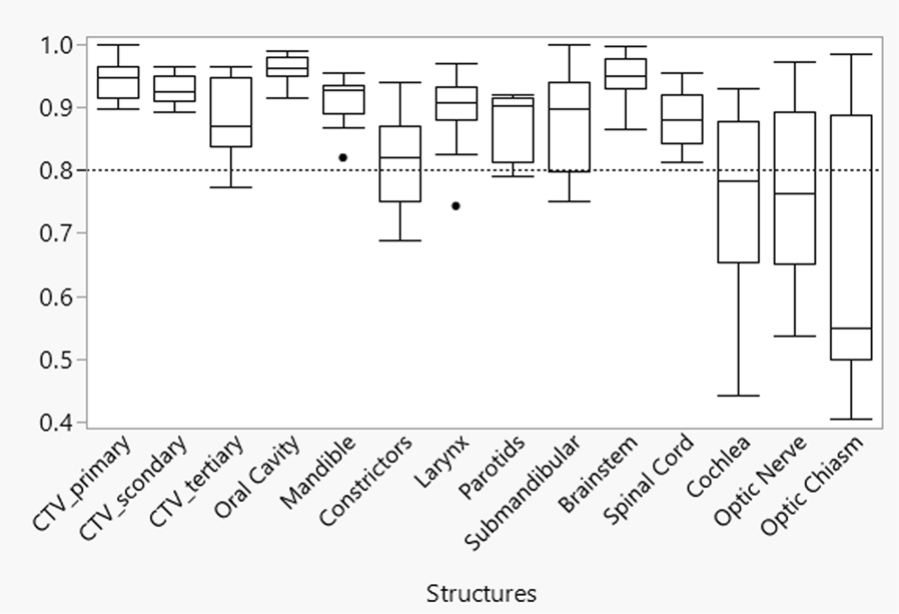
Box plot of dice similarity coefficient (DSC) for CTV and OARs. The dashed line is positioned at the recommended tolerance by AAPM report TG-132 (DSC > 0.8) [38]

**Fig. 4:**
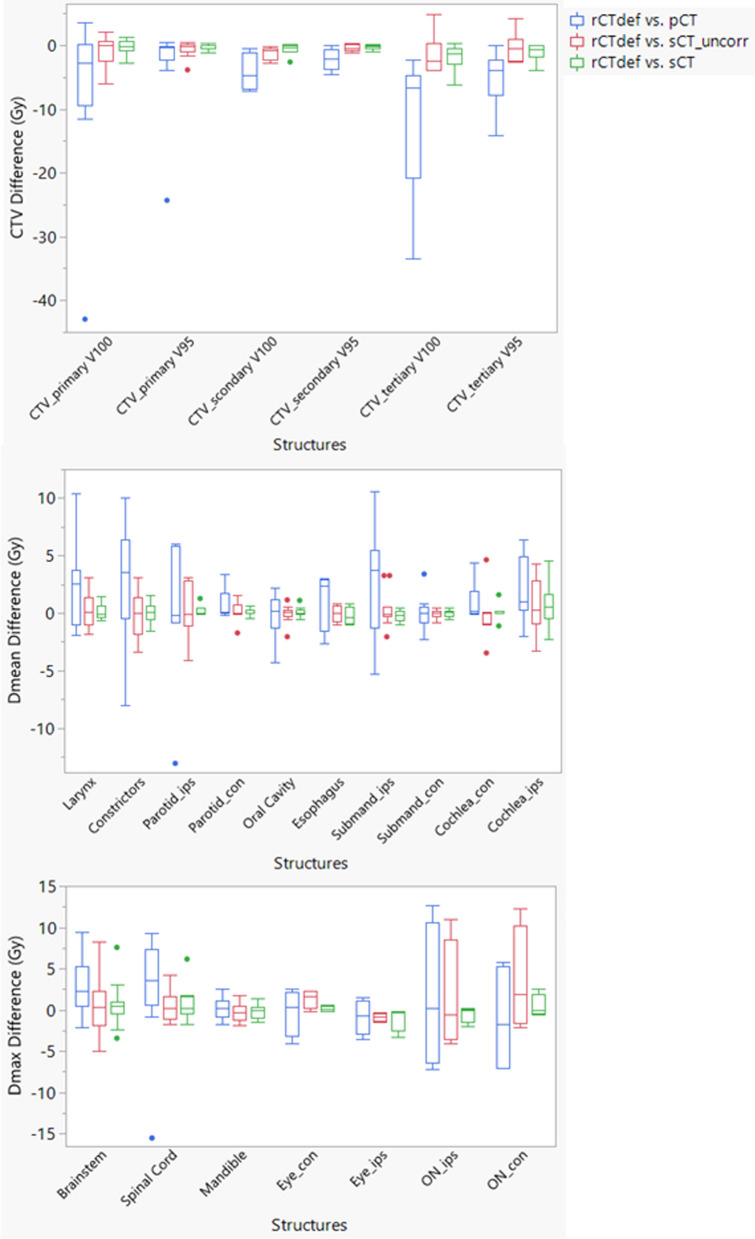
Box plots of the difference in dose-volume indices between rCT_def_ and pCT, sCT with physician-corrected contours, and sCT with uncorrected contours. The blue box represents the difference between rCT_def_ and pCT, the red box represents the difference between rCT_def_ and sCT with uncorrected contours, and the green box represents the difference between rCT_def_ and sCT with physician-corrected contours

The DSC for CTV and OARs between DIR-propagated contours and physician drawn contours were shown in Fig. 3. For the majority of cases (79%), the DIR-propagated contours have acceptable quality with a DCS > 0.8. However, for the smaller structures such as cochlea, manual correction was often required. The dose-volume indices obtained from rCT_def_, sCT with physician-corrected contours, and sCT_uncorr were also compared, and the results are shown in Table 3 and Fig. 4. With the uncorrected contours on the sCT for V100 estimation, the deviation range from the gold standard was (− 5.95%, 2.14%) for CTV_primary, (− 2.72%, − 0.22%) for CTV_secondary, and (− 3.9%, 4.8%) for CTV_tertiary. For CTV V95, the deviation range was (− 2.54%, 0.41%) for CTV_primary, (− 1.17%, 0.37%) for CTV_secondary, and (− 2.58%, 4.24%) for CTV_tertiary. Large deviations were observed when using the uncorrected contours to estimate the relative Dmean in OARs. Figure 4 shows that the maximum deviation of the relative Dmean for the larynx was (− 2.71%, 4.66%), for the constrictor was (− 4.85%, 4.42%), for the ipsilateral parotid (Parotid_ips) was (− 5.88%, 4.44%), and for the ipsilateral submandibular (Submand_ips) was (− 3.43%, 6.57%).

**Table 3:** Average difference of dose-volume indices between dose calculated on rCT_def_, pCT, and sCT with physician-corrected and uncorrected contours (range is reported in brackets)

	rCT_def_—pCT	*P* value	rCT_def_–sCT_uncorr	*P* value	rCT_def_—sCT	*P* value
Dmax (%), n = 13	0.16 ± 1.59	0.723	− 0.05 ± 1.41	0.902	0.02 ± 1.42	0.955
	(− 3.16, 2.47)		(− 2.41, 3.7)		(− 2.5, 3.71)	
Brainstem Dmax (%), n = 13	4.03 ± 5.19	**0.018**	0.74 ± 5.23	0.595	0.94 ± 3.98	0.402
	(− 2.99, 14.36)		(− 7.61, 12.49)		(− 5.23, 11.47)	
Cochlea_ips Dmean (%), n = 13	3.32 ± 4.45	**0.017**	0.93 ± 3.42	0.336	0.92 ± 2.83	0.277
	(− 2.91, 12.85)		(− 5.45, 6.51)		(− 3.28, 7.53)	
Cochlea_con Dmean (%), n = 9	1.49 ± 2.21	*0.078*	− 0.05 ± 2.99	0.959	0.16 ± 0.98	0.649
	(− 0.09, 6.27)		(− 4.95, 6.63)		(− 1.59, 2.28)	
Constrictors Dmean (%), n = 13	3.97 ± 7.31	*0.094*	− 0.33 ± 2.9	0.7	0.06 ± 1.22	0.919
	(− 11.49, 14.28)		(− 4.85, 4.42)		(− 2.37, 2.19)	
Larynx Dmean (%), n = 12	3.31 ± 5.29	*0.062*	0.49 ± 2.25	0.295	0.24 ± 1.02	0.376
	(− 3.2, 14.85)		(− 2.71, 4.66)		(− 0.9, 2.18)	
Mandible Dmax (%), n = 13	0.38 ± 1.95	0.536	− 0.47 ± 1.66	0.343	− 0.25 ± 1.24	0.491
	(− 2.56, 3.76)		(− 2.78, 2.59)		(− 2.23, 2.07)	
Oral Cavity Dmean (%), n = 13	− 0.27 ± 2.83	0.759	− 0.06 ± 1.1	0.881	0.14 ± 0.58	0.407
	(− 6.17, 3.07)		(− 3.11, 1.64)		(− 0.8, 1.57)	
Spinal Cord Dmax (%), n = 13	4.41 ± 9.38	0.135	0.68 ± 2.65	0.369	1.12 ± 2.92	0.194
	(− 22.21, 13.99)		(− 2.72, 6.48)		(− 2.73, 9.32)	
Parotid_ips Dmean (%), n = 8	− 0.29 ± 9.1	0.938	0.18 ± 3.52	0.886	0.39 ± 0.7	0.189
	(− 18.67, 8.57)		(− 5.88, 4.44)		(− 0.12, 1.82)	
Parotid_ips V20Gy (%), n = 8	0.75 ± 12.18	0.876	0.77 ± 5.35	0.717	1.17 ± 2.27	0.221
	(− 21.74, 18.85)		(− 9.17, 7.31)		(− 0.16, 6.26)	
Parotid_con Dmean (%), n = 10	1.11 ± 1.67	*0.066*	0.24 ± 1.24	0.564	0.13 ± 0.46	0.384
	(− 0.21, 4.87)		(− 2.46, 2.22)		(− 0.65, 0.95)	
Parotid_con V20Gy (%), n = 10	3.95 ± 3.02	**0.043**	− 0.08 ± 2.75	0.949	− 0.31 ± 1.3	0.618
	(− 0.07, 6.79)		(− 4.7, 2.52)		(− 2.12, 1.51)	
Submand_ips Dmean (%), n = 11	3.75 ± 7.16	0.129	0.65 ± 2.74	0.47	− 0.3 ± 0.79	0.208
	(− 7.55, 17.71)		(− 3.43, 6.57)		(− 1.51, 0.81)	
Submand_con Dmean (%), n = 10	0.09 ± 2.12	0.908	− 0.03 ± 0.57	0.817	0.03 ± 0.43	0.875
	(− 3.26, 4.85)		(− 1.21, 0.72)		(− 0.81, 0.7)	
Esophagus Dmean (%), n = 4	1.83 ± 3.78	0.953	− 0.05 ± 1.06	0.936	− 0.34 ± 1.11	0.589
	(− 3.76, 4.26)		(− 1.38, 1.2)		(− 1.38, 1.13)	
Eye_ips Dmax (%), n = 4	− 1.2 ± 3.03	0.487	− 1.28 ± 0.68	**0.035**	− 1.53 ± 2.26	0.262
	(− 5.08, 2.17)		(− 2.04, − 0.42)		(− 4.92, − 0.36)	
Eye_con Dmax (%), n = 4	− 0.24 ± 4.1	0.892	1.97 ± 1.65	*0.097*	0.18 ± 0.47	0.519
	(− 5.81, 3.78)		(− 0.35, 3.31)		(− 0.32, 0.82)	
ON_ips Dmax (%), n = 4	2.37 ± 13.22	0.764	2.3 ± 10.02	0.695	− 0.75 ± 1.56	0.406
	(− 10.28, 19.24)		(− 5.89, 16.67)		(− 3.09, 0.23)	
ON_cont Dmax (%), n = 4	− 1.59 ± 9.91	0.749	5.21 ± 9.56	0.355	0.7 ± 2.17	0.576
	(− 10.17, 8.74)		(− 3.05, 18.57)		(− 0.79, 3.92)	
Optic Chiasm Dmax (%), n = 4	6.49 ± 10.28	0.294	2.81 ± 5.1	0.355	2.91 ± 6.01	0.404
	(− 3.28, 19.82)		(− 3.73, 8.69)		(− 0.4, 11.92)	
CTV_primary V100 (%), n = 13	− 6.37 ± 11.98	*0.079*	− 0.86 ± 2.35	0.214	− 0.21 ± 1.18	0.528
	(− 42.96, 3.57)		(− 5.95, 2.14)		(− 2.72, 1.26)	
CTV_primary V95 (%), n = 13	− 2.72 ± 6.62	**0.036**	− 0.69 ± 1.33	0.154	− 0.2 ± 0.41	0.193
	(− 24.32, 0.46)		(− 2.54, 0.41)		(− 1.09, 0.29)	
CTV_scondary V100 (%), n = 7	− 4.23 ± 2.96	**0.018**	− 1.18 ± 0.98	**0.032**	− 0.46 ± 0.62	0.224
	(− 7.09, − 0.56)		(− 2.72, − 0.22)		(− 1.62, 0.18)	
CTV_scondary V95 (%), n = 7	− 2.17 ± 1.71	**0.027**	− 0.44 ± 0.56	0.116	− 0.25 ± 0.39	0.174
	(− 4.54, 0.07)		(− 1.17, 0.37)		(− 0.98, 0.14)	
CTV_tertiary V100 (%), n = 7	− 11.84 ± 11.64	**0.055**	− 1.52 ± 3.25	0.305	− 1.88 ± 2.25	*0.097*
	(− 33.5, − 2.25)		(− 3.9, 4.8)		(− 6.2, 0.33)	
CTV_tertiary V95 (%), n = 7	− 5.08 ± 4.78	**0.048**	− 0.31 ± 2.47	0.769	− 1.09 ± 1.47	0.13
	(− 14.1, − 0.07)		(− 2.58, 4.24)		(− 3.97, − 0.02)	

The number of structures included in the analysis are indicated in the first column as n. It should be noted that some structures were excluded from analysis as they were far from CTVs and received a cumulative dose < 1 Gy. The maximum dose (Dmax) and mean dose (Dmean) are reported as relative dose. A *P* value < 0.05 is highlighted in bold and indicates statistical significance. A *P* value < 0.1 is highlighted in italic

The physician-corrected contours on the sCT reduced the deviation from the gold standard, as expected. Table 3 shows that there was no statistical difference between the gold standard rCT_def_ and the sCT with physician-corrected contours (except for the tertiary CTV as described below). The deviation range of the relative Dmean was (− 0.9%, 2.18%) for the larynx, (− 2.37%, 2.19%) for the constrictor, (− 0.12%, 1.82%) for the Parotid_ips, (− 1.51%, 0.81%) for the Submand_ips, and (− 3.28%, 7.53%) for the ipsilateral cochlea. The maximum deviation of V100 and V95 for both CTV_primary and CTV_secondary was reduced to within 3% and 1% by using corrected contours. It should be noted that for some patients, part of the CTV_tertiary was out of the CBCT FOV when if it extended inferiorly to shoulder region. In these cases, the images out of the CBCT FOV were directly copied from the pCT to sCT. The deviation range for CTV_tertiary V100 and V95 was (− 6.2%, 0.33%) and (− 3.97%, − 0.02%), even with the corrected contour. As a result, it should be noted that it can be error prone to use the sCT to estimate the coverage of CTV_tertiary when it extends inferiorly. For the maximum dose to each OARs, the sCT without contour correction can produce large deviations for brainstem and optic nerves (ON). After the correction of contours, the deviation was smaller as compared to non-corrected contours for most OAR Dmax except ipsilateral eye.

### Adaptation versus non-adaptation

**Fig. 5:**
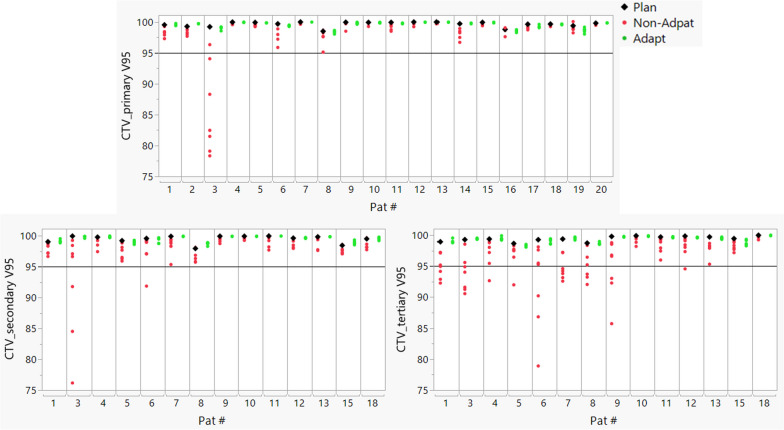
Dose-volume indices (V95) of planned dose (black diamond), non-adapted fractional dose (red dots), and adapted fractional dose (green dots) for the CTVs. A reference line is placed at V95 = 95%. Note that some patients only had a CTV_primary

**Fig. 6:**
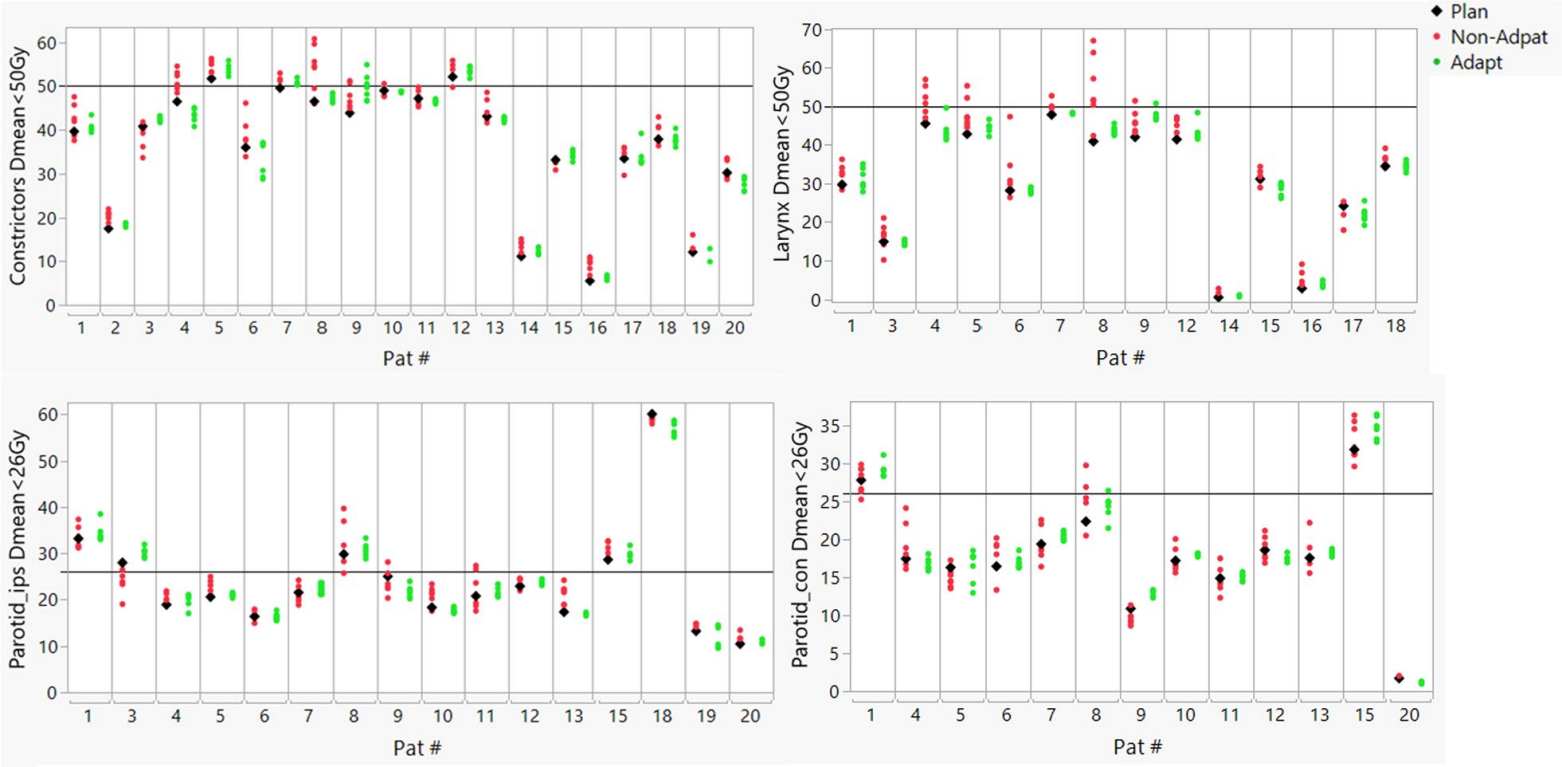
Dose-volume indices of planned dose (black diamond), non-adapted fractional dose (red dots), and adapted fractional dose (green dots) for constrictor, larynx, and bilateral parotid. A reference line is placed at constraint level for each OAR. Note that the figure does not present the dose-volume indices for some patients when the dose was too low (< 1 Gy) or when the parotid or larynx was the primary target

The fluctuation of weekly coverage for the CTVs and OAR dose-volume indices with or without adaptation can be found in Figs. 5 and 6, respectively. Without plan adaptation, 6 of 134 fractions (4.5%) for CTV_primary, 4 of 97 fractions (4.1%) for CTV_secondary and 26 of 97 fractions (26.8%) for CTV_tertiary failed to meet the dose constraints of V95 > 95%. In the adaptive cohort, the coverage constraints were met for all the fractions. The percentage of fractions failing to meet the dose constraint was reduced from 23.3% (30 of 129) to 17.8% (23 of 129) for constrictors and from 19.3% (16 of 83) to 1.2% (1 of 83) for larynx when plan adaptation was applied.

Table 4 compares dose-volume indices as well as NTCPs obtained from the planned dose, non-adapt accumulated dose, and adapt accumulated dose. Compared to the initial planned dose, the non-Adapt accumulated dose significantly increased the ipsilateral cochlea Dmean (1.17 Gy ± 0.8 Gy), the constrictor Dmean (1.75 Gy ± 2.3 Gy), the larynx Dmean (3.26 Gy ± 3.71 Gy), and the Parotid_ips V20Gy (2.72% ± 4%). The Dmax for the body (− 0.97% ± 1.45%) and mandible (− 0.8 Gy ± 0.54 Gy) were reduced. However, the coverage for all the CTVs decreased, especially for CTV_tertiary V100 (− 8.65% ± 6.99%). In the non-Adapt cohort, only 5 out of the 20 CTV_primary, 5 out of 14 CTV_secondary, and 3 out of 14 CTV_tertiary met the V100 > 95%. The V100 of CTV_primary, CTV_secondary, and CTV_tertiary dropped under 90% for 5, 2 and 7 patients, respectively. When plan adaptation was applied, all patients achieved V100 > 95% for all CTVs. For the OARs, the Adapt group plans significantly reduced the mean dose to the larynx (− 1.42 Gy ± 2.79 Gy) and constrictor (− 1.42 Gy ± 2.79 Gy) as compared to the non-adapt group. Plan adaptation did not show statistically significant benefits for any other OAR.

**Table 4:** Average dose-volume indices and NTCP for planned, non-adapted dose accumulation (non-adapt), and adapted dose accumulation (adapt)

	Plan	Non-adpat	Adapt	Non-adpat versus plan	*P* value	Adapt versus non-adapt	*P* value
Dmax (%)	109.09 ± 2.57	108.13 ± 2.15	107.88 ± 2.53	− 0.97 ± 1.45	**0.008**	− 0.24 ± 1.71	0.533
Brainstem Dmax (Gy)	25.21 ± 15.19	25.69 ± 15.01	24.21 ± 15.63	0.48 ± 1.35	0.131	− 1.47 ± 3.15	*0.050*
Cochlea_ips Dmean (Gy)	11.74 ± 8.3	12.91 ± 8.38	12.08 ± 8.77	1.17 ± 0.8	**0.034**	− 0.83 ± 0.9	*0.086*
Cochlea_con Dmean (Gy)	8.11 ± 8.3	8.13 ± 8.38	8.37 ± 8.77	0.02 ± 0.8	0.917	0.24 ± 0.9	0.369
Constrictors Dmean (Gy)	36.33 ± 14.25	38.08 ± 14.24	36.66 ± 14.55	1.75 ± 2.3	**0.003**	− 1.42 ± 2.79	**0.035**
Larynx Dmean (Gy)	28.45 ± 16.62	31.71 ± 18.61	29.13 ± 17.23	3.26 ± 3.71	**0.004**	− 2.58 ± 3.09	**0.006**
Mandible Dmax (Gy)	68.94 ± 3.98	68.14 ± 4.03	68.33 ± 3.91	− 0.8 ± 0.54	** < 0.001**	0.19 ± 0.97	0.398
Oral Cavity Dmean (Gy)	16.83 ± 12.72	16.74 ± 12.53	17.04 ± 12.72	− 0.09 ± 1.15	0.727	0.31 ± 1.13	0.24
Spinal Cord Dmax (Gy)	27.52 ± 12.54	27.78 ± 11.79	27.11 ± 12.58	0.26 ± 4.07	0.775	− 0.67 ± 4.13	0.476
Parotid_ips Dmean (Gy)	24.08 ± 11.39	24.89 ± 10.99	24.24 ± 11.1	0.82 ± 2.21	0.16	− 0.65 ± 2.28	0.272
Parotid_ips V20Gy (%)	46.21 ± 20.96	48.94 ± 21.31	47.83 ± 22.19	2.72 ± 4	**0.016**	− 1.11 ± 4.08	0.294
Parotid_con Dmean (Gy)	16.62 ± 8.45	16.94 ± 8.79	17.21 ± 9.02	0.32 ± 1.06	0.278	0.27 ± 1.56	0.523
Parotid_con V20Gy (%)	34.4 ± 19.37	35 ± 20.28	35.86 ± 20.92	0.6 ± 2.81	0.44	0.87 ± 3.85	0.414
Submand_ips Dmean (Gy)	37.42 ± 24.47	37.96 ± 24.18	37.69 ± 24.1	0.54 ± 1.94	0.457	− 0.27 ± 3.47	0.834
Submand_con Dmean (Gy)	35.34 ± 19.01	36.64 ± 19.42	35.1 ± 18.66	1.3 ± 2.35	*0.096*	− 1.55 ± 2.51	*0.068*
Esophagus Dmean (Gy)	36.48 ± 6.91	37.78 ± 5.61	36.59 ± 6.94	1.3 ± 1.65	0.11	− 1.2 ± 1.94	0.191
Eye_ips Dmax (Gy)	32.09 ± 17.48	31.01 ± 17.8	31.72 ± 17.11	− 1.08 ± 1.66	0.284	0.71 ± 0.87	0.201
Eye_con Dmax (Gy)	14.08 ± 8.13	13.79 ± 6.15	13.44 ± 6.05	− 0.3 ± 2.37	0.818	− 0.35 ± 2.2	0.770
ON_ips Dmax (Gy)	32.77 ± 11.33	33.07 ± 11.59	32.34 ± 11.22	0.3 ± 2.25	0.804	− 0.73 ± 2.48	0.597
ON_cont Dmax (Gy)	21.81 ± 10.86	20.79 ± 9.75	19.48 ± 10.97	− 1.02 ± 1.59	0.289	− 1.31 ± 1.73	0.229
Optic Chiasm Dmax (Gy)	15.59 ± 7.06	15.9 ± 8.11	14.68 ± 6.7	0.32 ± 1.08	0.597	− 1.22 ± 1.78	0.262
CTV_primary V100 (%)	95.15 ± 0.47	90.14 ± 7.94	96.3 ± 1.02	− 5.01 ± 7.87	**0.011**	6.16 ± 7.77	**0.002**
CTV_primary V95 (%)	99.44 ± 1.07	98.46 ± 3.5	99.3 ± 1.19	− 0.98 ± 3.28	**0.198**	0.84 ± 3.23	0.257
CTV_scondary V100 (%)	96.34 ± 2.25	92.31 ± 5.92	97.26 ± 1.51	− 4.03 ± 5.46	**0.016**	4.95 ± 5.87	**0.008**
CTV_scondary V95 (%)	99.47 ± 0.62	98.86 ± 0.79	99.41 ± 0.59	− 0.61 ± 0.41	** < 0.001**	0.56 ± 0.38	** < 0.001**
CTV_tertiary V100 (%)	96.33 ± 2.32	87.68 ± 8.13	96.96 ± 1.4	− 8.65 ± 6.99	**0.001**	9.28 ± 7.64	**0.001**
CTV_tertiary V95 (%)	99.42 ± 0.43	96.71 ± 3.18	99.2 ± 0.51	− 2.71 ± 3.08	**0.006**	2.49 ± 3.06	**0.009**
NTCP Dysphagia (%)	6.69 ± 6.23	8.68 ± 8.94	7.28 ± 7.19	1.99 ± 3.66	**0.025**	− 1.4 ± 3.55	*0.093*
NTCP Larynx Edema (%)	16.26 ± 21.41	26.05 ± 33.95	18.53 ± 24.3	9.79 ± 15.75	**0.021**	− 7.52 ± 13.59	**0.037**
NTCP Xerostomia_ips (%)	19.53 ± 20.46	20.63 ± 20.28	20.06 ± 20.02	1.1 ± 3.49	0.228	− 0.56 ± 3.22	0.495
NTCP Xerostomia_con (%)	7.37 ± 8.18	7.69 ± 8.54	8 ± 9.4	0.32 ± 1	0.171	0.31 ± 1.57	0.387
NTCP Esophagus (%)	22.48 ± 17.32	24.42 ± 17.49	23.1 ± 17.57	1.94 ± 2.46	*0.061*	− 1.31 ± 2.9	0.241

A *P* value < 0.05 is highlighted in bold and indicates statistical significance. A *P* value < 0.1 is highlighted in italic

**Fig. 7:**
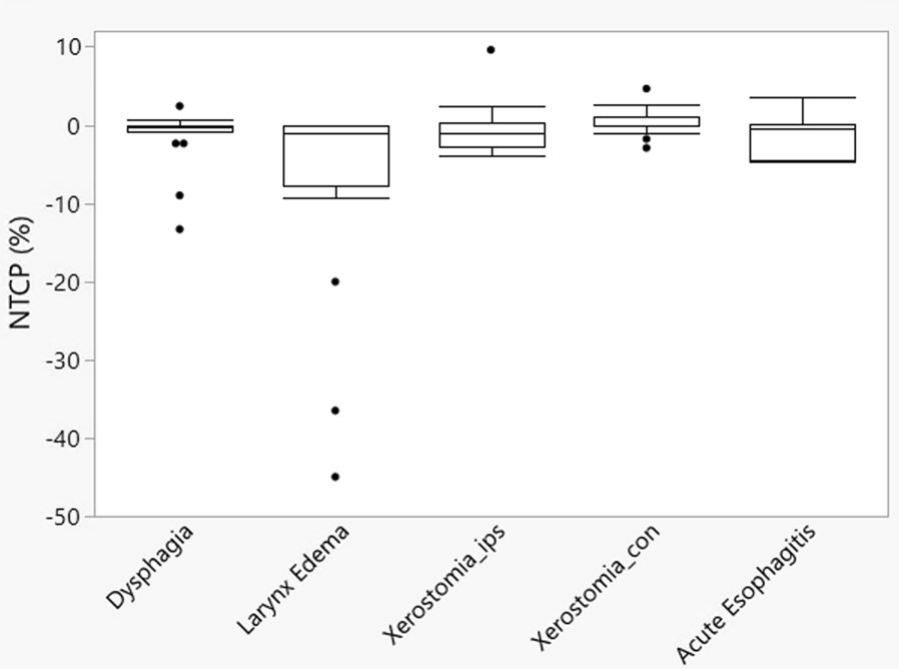
Box plots of NTCP difference between the adapt and non-adapt cohorts

Figure 7 shows the difference in NTCP between adapt and non-adapt groups. It was notable that the NTCP of larynx edema was significantly reduced by 7.52% ± 13.59% in the adapt cohort as compared to the non-adapt cohort, while achieving a maximum reduction of 45% for patient 8 with significant weight loss, as shown in Fig. 7. For other structures, there was no statistically significant difference in NTCP between the adapt and non-adapt groups.

**Fig. 8:**
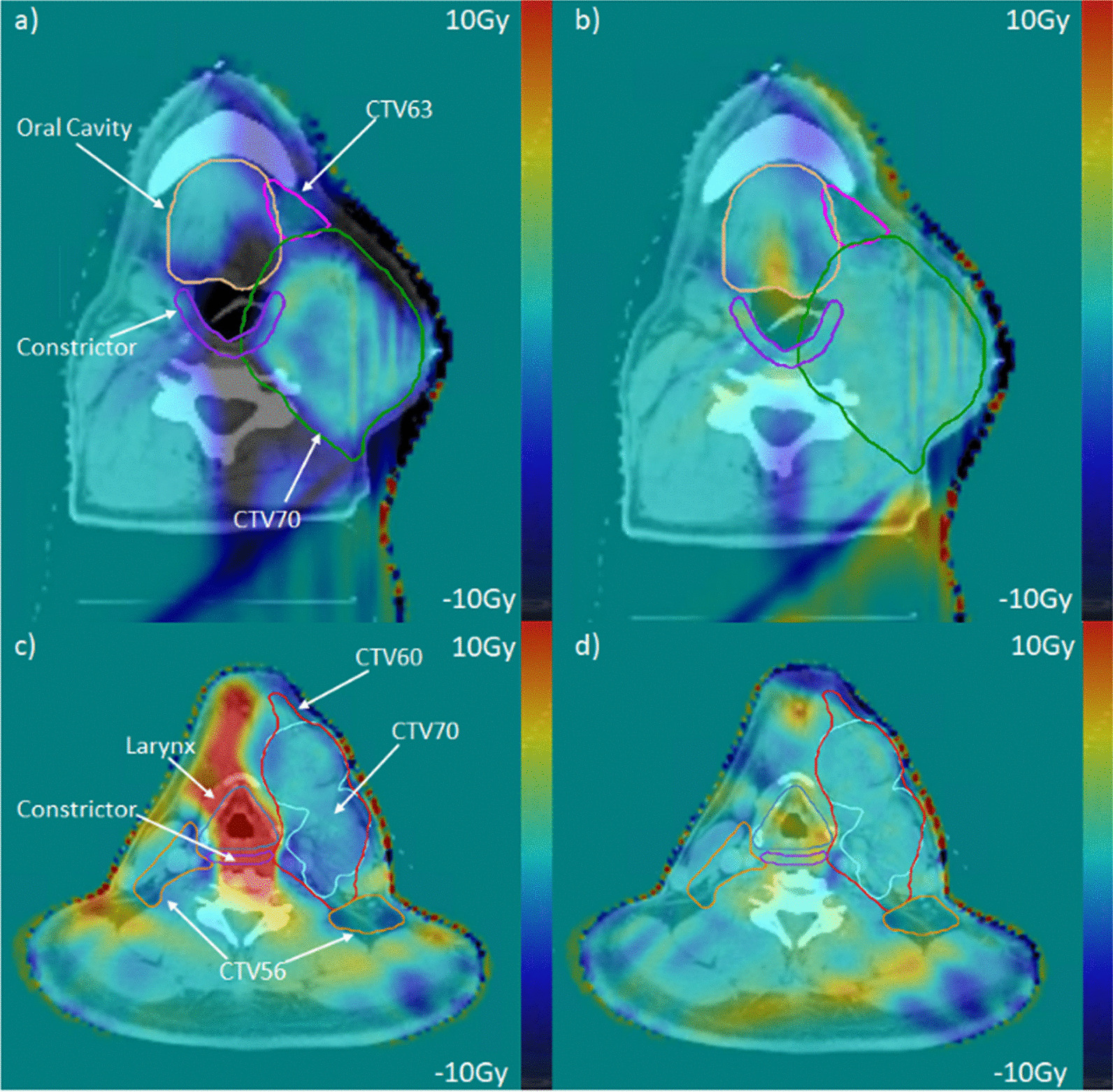
Dose difference map for patient 3 (**a** and **b**) and patient 8 (**c** and **d**). The left column shows the difference between planned and non-adapted dose and the right column shows the difference between the planned and adapted dose

An example case (Patient 3) with a large tumor growth is illustrated in Fig. 8. With the large tumor growth, the non-Adapt accumulated dose showed considerable under dosage to CTV and OARs as compared to the planned dose (Fig. 8a). With the plan adaptation, accumulated dose was comparable to the planned dose, as shown in Fig. 8b. Another example (Patient 8) is shown in Fig. 8c. This patient encountered significant weight loss during treatment course. The Non-Adapt accumulated dose greatly increased the dose to the larynx and constrictors, as shown in Fig. 8c, while the Adapt accumulated dose resulted in reduction in dose to the OARs with a comparable dose distribution to the planning dose (Fig. 8d). The mean dose to the larynx, constrictor, ipsilateral parotid, and contralateral parotid increased by 14.13 Gy, 7.75 Gy, 3.69 Gy and 2.52 Gy, while the plan adaptation reduced the mean dose by 11.08 Gy, 7.31 Gy, 1.02 Gy and 1.35 Gy, respectively.

## Discussion

The use of sCTs for proton dose calculation based on various methods has been explored by several groups [10–15]. Thummerer et al. compared proton dose calculation with corrected CBCT using several methods and concluded that accurate proton dose calculation can be achieved with both DIR-based and deep learning-based sCT in HN region [19]. The high gamma passing rate for this study between dose calculated on sCT and the gold standard demonstrates that the DIR-based sCT provides accurate SP information for proton dose calculation, which was consistent with other studies [10–15]. Although we assumed CBCT and rCT had the same anatomical information, there were some discrepancies in cavity filling or emptying observed between the CBCT and rCT_def_ for some patients due to changes in the tongue position. To mitigate its impact, the field-specific targets prevented beams entering through the chin and teeth area.

Lack of a robust automatic contour propagation method was still an obstacle for online adaptation. We found that the DIR-propagated contours achieved acceptable quality as compared to gold standard contours (DCS > 0.8) for most structures while more attention should be paid to constrictors and small structures (e.g., cochlea). The dosimetric difference between rCT_def_ and pCT represented the deviation caused by anatomical changes and setup error, while the difference between rCT_def_ and sCT with corrected contours was due to the SP transfer uncertainty arising from DIR inaccuracy. We found that the dose deviation caused by anatomical changes and setup error was much higher than the deviation caused by DIR inaccuracy. The errors introduced by using sCT to estimate the dose-volume indices can be further reduced by using physician-corrected contours for most structures, which is consistent with the previous findings [15]. It was observed that for structures proximal to CTV (e.g., ipsilateral parotid), the contour correction by physician was more impactful to the DVH evaluation. Estimation of the dose-volume indices for small structures (e.g., cochlea), even with corrected contours, was still unreliable. Although using the uncorrected contours on sCT to estimate CTV coverage can potentially yield acceptable accuracy, it was still beneficial to perform contour corrections for the CTVs. The CTV was one of the most crucial structures and required careful attention. Of note, the CTV_tertiary was typically out of CBCT FOV when it extended inferiorly to the shoulder region. In these cases, the corresponding information from the pCT was filled in and could lead to possible overestimations of the CTV coverage. An extended FOV CBCT scan to include the shoulder region is recommended to improve the accuracy of dose evaluation for CTV tertiary based on sCT. It should be acknowledged that relying solely on a simple DVH comparison may overlook significant dose differences, particularly in small volume regions near the end of the treatment range.

A study by Kurz et al. investigated sCT with DIR generated contours for HN replanning and found improvement of plan quality with significant hotspot reduction and partial improvements in OAR sparing [20]. Another group investigated the use of uncorrected contours on sCT for replanning of lung patients and indicated that this approach can still restore the CTV coverage and reduce the hotspots [35]. Therefore, in the context of using CT images for dose evaluation or replanning, whether with or without corrected contours, it is crucial to have a clear understanding of the associated uncertainties, as highlighted in the study. One of the limitations of this study was the small sample we included for the validation of sCT for proton dose calculation (n = 13).

In the present work we found that the constrictors and larynx significantly benefited from APT. The improved target coverage was a common finding in previous studies looking into the benefits of APT [20–22, 25, 26]. Gora et al. explored the benefits using six HN patients and found that the APT reduced the hotspots in brainstem by 8 Gy and spinal cord by 14 Gy [25]. The current study included a larger cohort of patients with CTVs in the mid/lower neck area for the majority of patients. However, there were no significant differences between non-Adapt accumulated Dmax and Adapt accumulated Dmax or planned Dmax for spinal cord and brainstem. It was worth noting that the benefits of adaptive proton therapy can be largely patient dependent. In this study, it was observed that patients who underwent weight loss can suffer from extreme overdosage to OARs (e.g., patient 8).

The NTCP models employed reveal that plan adaptation would significantly reduce the probability of larynx edema. 30% of the patients would benefit from APT with reduction of larynx edema by more than 5%, including the maximum reduction of 44.97%. For dysphagia, 10% of the patients achieved reduction of NTCP by more than 5% using adaptive proton therapy, with the maximum reduction of 13.33%. For xerostomia and acute esophagitis, adaptation did not provide an NTCP benefit of more than 3% compared to non-Adapt for all patients. For some patients, in order to restore target coverage, plan adaptation increased the OAR dose (e.g., patient 2), and hence an increase in NTCP was acceptable. It should be noted that the NTCP value could be improved if the optimizer was given more flexibility during re-optimization, this can be minimized by employing the automated planning model for objective list generation.

In this study, we applied the setup uncertainty during re-optimization. It might be reasonable to assume there was no setup error for HN patient with online adaption, which would provide further OAR sparing [22]. While our previous work demonstrated that the weekly sampling was sufficient to represent the total delivered dose, it should be acknowledged that it was one of the limitations of this study [3]. It might be was possible that weekly sampling could miss weight loss occurring in a short period of time. Additionally, dose accumulation error could be feathered out if more fractions were employed [36].

In this work, a validated proton-specific automated planning system was employed for both initial planning and re-optimization. It was shown in our previous study that the trained automated planning model can generate IMPT plans comparable to expert plans [28]. Manual full re-optimization might be the best option to regain the optimal dose distribution, but it was only applied for offline adaptation. Another option for adaption was to use the initial objective list for re-optimization, but there was a risk of not achieving the optimal dose distribution with significant anatomical differences. Fast dose restoration methods have been investigated by several groups for online adaptive proton therapy because of its high speed for plan adaptation and has been demonstrated to improve the plan quality compared to the non-adapted treatment [24, 26, 37]. Borderías-Villarroel et al. compared dose restoration and manual full plan optimization for plan adaptation and found that dose restoration avoided full optimization for 52% of patients but the remaining patients with a large anatomical change or inaccurate positioning still needed full offline optimization [23]. In this study, we employed an automated planning software for re-optimization first to diminish inter-operator variation during re-optimization and second to generate the optimal dose distribution. Since this was still a full re-optimization, the time required was around 15–20 min. However, compared to the manual re-optimization, which requires iteratively tuning the objective list and demands hours, utilizing the model substantially reduces the workload. sCT generation and contour propagation add an additional 5–10 min. With manual contour adjustment, 5–10 additional minutes were required, depending on the DIR quality and number of critical organs required for manual adjustment. In its current form, this method is feasible for offline adaptation, but application to online adaptive proton therapy still requires additional validation including investigation of a fast online QA procedure. Nevertheless, we did observe the benefits of employing this model for restoration of CTV coverage and OAR sparing.

## Conclusions

This study assessed the feasibility and potential benefits of CBCT-based adaptive proton planning using an automated planning software for treatment of HN cancer. CBCT-based sCT was demonstrated to be a powerful tool for accurate proton dose calculation in adaptive proton therapy. Contour correction is recommended to reduce the uncertainty for dosimetric parameter estimation, especially for OARs with relatively small volumes. It was found that adaptive IMPT using automated planning of HN cancer resulted in better target coverage and sparing for larynx and constrictor OARs compared to non-adaptation. The NTCP of larynx edema was significantly reduced compared with non-adaptive IMPT, but the magnitude of potential benefits from APT were patient dependent.

## Data Availability

All references are cited in the manuscript. There is no additional data and material involved.
